# Preventing Youth Crime and Violence: Intervention and Evaluation Issues

**DOI:** 10.3390/bs16020247

**Published:** 2026-02-09

**Authors:** Nick Axford, Sajid Humayun

**Affiliations:** 1 Peninsula Medical School, University of Plymouth, Plymouth PL6 8BU, UK; 2 School of Human Sciences, University of Greenwich, London SE10 9LS, UK; s.humayun@greenwich.ac.uk

**Keywords:** crime, evaluation, prevention, violence, youth

## Abstract

Whilst youth offending has been declining, there have been increases in serious youth violence in the last decade. Therefore, there is a pressing need to prevent youth crime and violence owing to its prevalence, harms and cost to society. Part of the effort to address this involves identifying and disseminating evidence-based practice. We explore key challenges in this endeavour and offer ideas for how to address them. These fall into two categories. The first concerns the focus and nature of interventions and the imperative to increase the effectiveness of our collective efforts. We start by considering neglected issues and groups in need of intervention responses, arguing that interventions too often do not consider relevant risk and contextual factors. Next, we explore emerging means of designing and delivering interventions that warrant greater investment, including those that extend beyond a traditional focus on programmes. Finally, we highlight cross-cutting issues affecting the delivery and uptake of interventions and therefore their success. The second set of challenges concerns intervention evaluation and the need to maximise the usefulness of our cumulative evaluation activity in this field. Here, we start by discussing common challenges involved in moving through the pipeline of feasibility, pilot and definitive (often trial-based) evaluations. We then explore issues concerning the actual design and conduct of such studies, before closing with thoughts on the potential value of underused (non-trial) methods of impact evaluation. Throughout the article, we draw on the scientific literature and our collective experience over many years of developing, adapting, evaluating and promoting interventions and other forms of evidence-based practice in this space.

## 1. Introduction

There is a pressing need to prevent youth crime and violence owing to its prevalence, harms and cost to society (Kieselbach & Butchart, 2015). In the year ending March 2022, there were 33,000 proven offences committed by children and young people (CYP) in England and Wales. Around 8000 CYP were first-time entrants to the criminal justice system, while 3500 proven knife and offensive weapon offences were committed by CYP (Youth Justice Board for England and Wales, 2023). Whilst youth offending has been declining across many countries over the last ten years (e.g., Polglase & Lambie, 2024), recent data show that serious violence affecting CYP in England and Wales is higher than it was a decade ago. Black CYP, boys and those living in the poorest areas are disproportionately affected, with boys from Black backgrounds being much more likely to be cautioned or convicted for violence in the UK, despite White CYP having more interaction with police and the justice system (Youth Endowment Fund, 2024). In particular, boys caught up in serious violence have poor life prospects; on average, they will gain fewer qualifications, earn less, suffer poorer mental and physical health, and die younger than their peers (Piquero et al., 2010, 2014). The financial cost of youth crime and violence is also significant: for example, the economic and social cost of serious youth violence (gun- or knife-related offences committed by CYP aged ≤ 24) across England and Wales in the period 2008–2019 was £11 billion (Irwin-Rogers et al., 2020).

Supporting CYP to avoid serious crime and violence is therefore one of the most critical social policy problems and is subject to heightened political and media interest in the UK following recent high-profile knife attacks and rioting involving CYP. Part of the effort to address this involves identifying and disseminating evidence-based practice. Much is known about the effectiveness of school-, family- and community-based interventions to prevent and address youth crime and violence (e.g., Matjasko et al., 2012; Fagan & Catalano, 2013; Farrington et al., 2017; Russell, 2021; Kovalenko et al., 2022). Many of these are included in evidence-based programme (EBP) clearinghouses such as Blueprints in the US, the Foundations Guidebook in the UK and the Europe-wide Xchange.

There have been significant efforts to increase the supply of these and new interventions, most notably in the UK through the Youth Endowment Fund (YEF), established in 2019 with a £200 million endowment and a 10-year mandate from the Home Office. The YEF strategy includes funding new evaluations to help fill gaps in the evidence and publishing guidance on how to change practice accordingly. In 2022, with funding from the YEF and a major philanthropist, the Ending Youth Violence Lab launched with a commitment to build a pipeline of high-quality, UK-based interventions that are ready for—and committed to—rigorous impact evaluation. It does this through the development and early-stage testing of interventions.

To inform the Lab’s strategy, we were invited (independently) to advise how it could best deliver on its mission. We were asked to consider promising interventions and approaches the Lab should pursue; methodological approaches to lay the groundwork for high-quality trials and more positive results; key factors to consider in adapting programmes from overseas and designing and delivering feasibility and pilot evaluations; and other relevant issues. Our respective think pieces informed an ‘evidence manifesto’ (Ending Youth Violence Lab, 2024). In this opinion article, we distil, discuss and synthesise these documents, discussing what, in our judgement, are key challenges involved in the endeavour to promote evidence-based policy and practice initiatives to prevent and reduce youth crime and violence, and offering ideas for how to address them. These fall into two categories: the focus and nature of interventions, and approaches to evaluation. This reflects the need, as we see it, to increase the effectiveness of our collective intervention efforts in the field and to maximise the usefulness of our cumulative evaluation activity.

We draw on the scientific literature, including YEF-funded studies, and our collective experience over many years of developing, adapting, evaluating and promoting interventions and other forms of evidence-based practice in this space. NA has conducted randomised controlled trials (RCTs) of bullying prevention, parenting support and youth mentoring programmes, supported the adaptation of interventions across international contexts, and served on evidence panels for two programme registries. SH, meanwhile, has conducted RCTs of family therapy and parenting programmes for offending and child criminal exploitation (CCE) and served on evidence and advisory panels for UK What Works Centres. We draw primarily on the literature from England and Wales but also make use of the wider evidence base, particularly from the US.

## 2. Intervention Issues

In this section, we first discuss neglected issues and groups in need of intervention responses, arguing that interventions too often do not consider relevant risk and contextual factors. Next, we explore emerging means of designing and delivering interventions that warrant greater investment, including those that extend beyond a traditional focus on programmes. Finally, we highlight cross-cutting issues affecting the delivery and uptake of interventions and therefore their success.

### 2.1. Factors That Increase Vulnerability to Crime and Violence

#### 2.1.1. Callous–Unemotional Traits

The presence of callous–unemotional (CU) traits delineates a subgroup of antisocial CYP at particularly high risk of violence (Ribeiro da Silva et al., 2020) and lifetime criminality (Kahn et al., 2013). Traditionally, intervention approaches have been thought to be ineffective (Hawes & Dadds, 2005). More recent research on associations between parenting and CU traits (Trentacosta et al., 2019) suggests that adapting existing parenting interventions may lead to improved outcomes. However, there have been few recent RCTs (Dadds et al., 2019; Fleming et al., 2022), and additional feasibility work is needed to translate some of these primary research findings into viable intervention approaches. Another potentially promising avenue of clinical research is to explore whether particular subgroups of CYP with CU traits, supposedly reflecting different etiology (Karpman, 1941; Cecil et al., 2018), may be more amenable to treatment efforts (Fleming et al., 2023).

#### 2.1.2. Adverse Childhood Experiences

Research on the association between trauma and violence has typically focused on CYP maltreatment (Widom & Wilson, 2015), but it may be more sensible to consider adversity more broadly when attempting to identify at-risk CYP, such as exposure to community violence (e.g., Walsh et al., 2025), and potentially through the lens of latent vulnerability models (McCrory & Viding, 2015). For example, a review of the case histories of the most prolific young criminals in London found extensive histories of adverse childhood experiences (ACEs; Hilder et al., 2021). Similar findings exist in the UK for knife crime and gang activity (Haylock et al., 2020). Therefore, when attempting to target or screen for individuals who may be at most risk of violent behaviour, it may be sensible to attempt to identify a history of trauma rather than just focusing on current flags for risk of violence and antisocial behaviour. One way to do this may be to conduct more trials recruiting CYP known to child social care rather than those known to just Youth Justice Services or the broader criminal justice system, because those known to both youth offending services and social care appear to have more of a history of violent offending (Baidawi & Ball, 2023).

Given the associations between ACEs and the risk of violence and antisocial behaviour, trauma-informed intervention approaches are an important area for research efforts. There is increasing interest in the police engaging in a trauma-informed approach to CYP, with some interest lying in developing approaches to attempt to identify at-risk CYP at earlier stages to thereby facilitate prevention work.^1^ One potential approach involving the police is identifying CYP at a point of crisis/during a reachable moment. For example, joint working between the police and social care during police custody or when a CYP has been recovered by a service when missing from home may lead to CYP engagement in services when it would otherwise be rejected.^2^ However, it should be noted that there are still large gaps in understanding causal pathways amongst CYP experiencing the same kinds of adversity. Some research has been conducted on specific mechanisms by which adversity leads to offending, such as emotional dysregulation (Wolff & Baglivio, 2017) or cognitive dysfunction (Baer & Maschi, 2003). This, in turn, has led to the adaptation and use of intervention approaches such as Cognitive Behavioural Therapy (Zettler, 2021). However, further work is required to better inform the development of effective interventions, which in turn requires a better understanding of equifinality and developmental pathways amongst CYP experiencing adversity (Hawes & Allen, 2023).

#### 2.1.3. Financial and Material Disadvantage

Low income is often the context but rarely the focus of interventions seeking to prevent or address youth crime and violence. Social work with CYP also tends to overlook poverty—described as the “wallpaper of practice” in the sense of being “too big to tackle and too familiar to notice” (Morris et al., 2018, p. 370). This situation is not sustainable. Poverty has adverse causal effects on a range of CYP outcomes, including health, behaviour and educational performance (Cooper & Stewart, 2021). There is also a strong relationship between offending, justice contact and poverty in the teenage years and early adulthood, with poverty known to be directly related to involvement in youth violence (McAra & McVie, 2022). A recent review and meta-analysis did not find that poverty causes youth crime and violence, owing to the type of studies included and the difficulty of inferring causality, but did find that poverty is a risk factor for youth crime and violence (Clemmow et al., 2025b). The strongest links (small-bordering-moderate effect sizes) were for financial problems (e.g., food insecurity, debt, money worries) and low income. Further, low socio-economic status is associated with lower rates of attendance in psychosocial interventions such as parenting programmes, owing to practical constraints or feelings of stigma and shame (e.g., Berry et al., 2023).

Efforts to prevent or reduce youth violence and its precursors therefore need to pay greater attention to family financial well-being. Indeed, the evidence shows that improving family financial well-being can contribute to reducing ACEs and improving CYP psychosocial outcomes (Courtin et al., 2019; Cooper & Stewart, 2021). Clearly, social and economic policy—for instance regarding the living wage, taxation, benefits and employment—plays an important role, and there is evidence for its effectiveness (Wickham et al., 2016). However, there is also a case for local policy initiatives, such as supporting female employment and poverty-proofing the school day, and for innovation in frontline services, from integrating income maximisation into psychosocial interventions to promoting more poverty-aware practice (Axford & Berry, 2023). All such innovation needs testing for acceptability, implementation and impact, and interventions should continue to seek to address other well-evidenced psychosocial risk and protective factors.

#### 2.1.4. Child Criminal Exploitation and County Lines

There is increasing concern over CYP being drawn into violent criminal behaviour through child criminal exploitation (CCE), especially via gang activity and drug dealing. In particular, involvement in county lines drug networks (CLDNs) appears to be associated with heightened risk of violent victimisation (Black, 2020; Child Safeguarding Practice Review Panel, 2020). Many of these CYP will be victim–offenders, committing violence against other CYP themselves, and are at high risk of criminal conviction (Sturrock & Holmes, 2015), which, in turn, increases their vulnerability to subsequent exploitation. While some argue that involved CYP exert agency when engaging in CLDNs (Moyle, 2019), this is best understood in the context of the limited set of choices available to them within their social fields (Firmin, 2020), and evidence of clear coercion and control are common.

Many exploited CYP are known to social services, but effective practice for tackling involvement in CLDNs is rare (Child Safeguarding Practice Review Panel, 2020). This is partly due to limited understanding of the risk factors and mechanisms involved, thereby limiting the development of effective intervention approaches. However, some specific risk factors have been identified. Specifically, family breakdown, being missing from home, and school exclusion all appear to increase the risk of CLDN exploitation (ECPAT UK & Missing People, 2018; National Crime Agency, 2019; Child Safeguarding Practice Review Panel, 2020). Therefore, there is scope to develop new intervention approaches, or adapt existing ones, in order to reduce the risk of CCE and CLDN involvement. Given the adverse outcomes of these exploited CYP, there is a pressing need to conduct feasibility work in this area.

While CCE is likely to become an increasingly important route to youth crime and violence, almost nothing is known about which interventions might be effective. One approach is to attempt to adapt existing EBPs to target associated risk factors. The YEF has funded at least two trials attempting to do this—for Functional Family Therapy (FFT; Humayun et al., 2023) and Multisystemic Therapy (MST; Langdon et al., 2023)—with some success. Both approaches attempt to use the family as a protective mechanism to help CYP make safer choices in the face of contextual risk. This approach has some merit. However, given that contextual risk is extra-familial risk, interventions that have more of a focus on systems outside the family may also be effective.

Perhaps more pressing is the need to ensure that CYP at risk of CCE receive some support, even if those services are yet to be evaluated, because no further action is the most likely outcome when these CYP are referred to social care (Lloyd & Firmin, 2020). When there is a service response, it often involves moving the CYP out of borough, which, while potentially effective in the short term, is not a viable long-term strategy (Child Safeguarding Practice Review Panel, 2020). Instead, exploited CYP are more likely to be seen by youth offending services or the police, with a risk of criminalisation for drug offences. Firmin (2020) argues that these responses or lack of responses are part of a broader problem with UK CYP social work, which is designed to deal with familial, rather than extra-familial, harm (likely to also be a problem beyond the UK). Therefore, there is some merit in trialling system-level approaches that aim to change the way contextual risk is dealt with, with the aim of providing better services to these highly vulnerable CYP.

### 2.2. New Approaches to Designing and Delivering Interventions

#### 2.2.1. Teletherapy

One of the challenges to the sustainability of EBPs is cost. This naturally has an impact on how many families and CYP can be seen and is exacerbated in rural areas where practitioners may need to spend time travelling to sites or to family homes. The COVID-19 pandemic resulted in some evaluation of remote delivery or teletherapy, resulting in evidence that clinical outcomes were not adversely affected, even in the case of intensive approaches typically delivered in the family home, like FFT (Lange et al., 2023). Remote delivery allowed therapists to increase their caseload and thereby increase the number of families seen, as well as improving access to CYP and families. Thus, families who might not otherwise travel may be more likely to attend remote sessions, as might family members in different locations. These include CYP on devices in their own rooms who would otherwise refuse to engage in a joint session with their caregivers, or key family members who might be unable to attend because of travel for work. Furthermore, the benefits of teletherapy extend beyond family therapy interventions to group-based interventions, which can also be delivered entirely online (Whitchurch et al., 2022). Further investigation of the efficacy of online versions of established EBPs is therefore warranted.

#### 2.2.2. Place-Based Approaches

In isolation, programmes are limited in what they can achieve. There is a strong case for encouraging their implementation in the context of broader cross-sector coalitions (e.g., statutory, business, third sector) working strategically to address complex social problems (such as youth crime and violence). These include manualised initiatives such as Communities that Care (CTC), which promote community-wide investment in prevention and early intervention to address outcomes including youth crime and violence. A large cluster trial of CTC in the US found positive and sustained impacts on various outcomes related to youth crime and violence (e.g., Oesterle et al., 2018). Meanwhile, a broad range of place-based models for preventing youth violence also exist. These typically target multiple levels of influence and need, involve strong community engagement, and include activities such as youth development and parenting programmes, community mobilisation and school-based intervention (Baidawi et al., 2023). Evidence from mostly quasi-experimental designs suggests that these can have a positive impact on youth violence (Baidawi et al., 2023).

Such ‘collective impact’ initiatives provide an infrastructure for developing and implementing new interventions or better aligning existing services to address an identified need (Kania & Kramer, 2011). Done well, they create a strong sense of community ownership by involving commissioners, practitioners, service users and other members of the public. They identify community assets but also gaps in provision, which helps with prioritising action. ‘Backbone organisations’ coordinate the process and support service design and implementation. Approaches vary in terms of how structured they are and how much they focus on existing programmes or new initiatives. Case studies and theory-based evaluations of collective impact models demonstrate promising results (Lynn et al., 2018).

Inevitably, there are challenges involved in place-based and collective impact approaches. Criticisms include a failure to engage community members with lived experience, resulting in inappropriate solutions, and a danger that service delivery solutions supersede more radical policy and system reforms needed to address structural issues and achieve a transformative change in outcomes (Smart, 2017). Practically, it can be hard for participating organisations to align shared goals with their own, especially with stretched workloads and budgets, and there can be issues with data infrastructure and sharing (Carp & Lundy-Wagner, 2016). Meanwhile, the complex and dynamic nature of initiatives can make it difficult to coordinate evaluation activities and attribute outcomes (Panjwani et al., 2023). Addressing these will be necessary to support the success of such initiatives.

#### 2.2.3. Common Elements

EBPs can be expensive to implement well and often require stopping an existing practice to create capacity, which can be challenging politically and logistically. They may not fit into existing services because they are not ‘system ready’ or the system is not ‘programme ready’. Practitioners often find them too rigid, and implementation with fidelity is difficult. Perhaps in part for these reasons, few EBPs have been scaled.

This has prompted efforts to identify the common elements of EBPs and promote their use in practice (Embry & Biglan, 2008; Lipsey, 2020; Leijten et al., 2021). Elements may be categorised as (i) characteristics of interventions (e.g., duration intensity, delivery mode, targeting) or (ii) activities, practices or ‘units of behavioural influence’ (e.g., skill-building, problem-solving, time out). Methods for identifying them include coding interventions based on evaluations or programme manuals, consulting practitioners or service users familiar with the intervention, or conducting meta-regressions in the context of a meta-analysis to identify factors associated with larger effects (McKaskill et al., 2021).

There are several arguments for the common elements approach. It generalises key practices and principles that apply to a range of effective programmes, permits better informed programme adaptation and supports continuous improvement via the ability to integrate components into existing practice. Additionally, it supports the scaling of evidence-based practice, because it is easier than scaling up entire programmes, and can be used in interactions beyond structured sessions or formal contexts, whether involving professionals or volunteers (e.g., interactions with young people in sports coaching, or situations that occur between lessons in school). Of course, a common elements approach is not without criticism: for example, that it is less an intervention strategy than an analytic summary, since it is the combination and sequencing of elements that makes a difference to outcomes. Further, their use may require as much—if not more—implementation support as programmes.

Documented applications of common elements in practice are scant, however, and as are evaluations of their impact. There is therefore considerable scope to expand efforts to mobilise and test common elements in the pursuit of preventing and reducing youth crime and violence. For example, a current study is supporting multiple small youth mentoring providers to deliver a shared practice model based on core components (Lewis et al., 2023). Other subject areas that may merit a common elements approach owing to their substantial evidence base include parenting support and school-based interventions to prevent dating violence and bullying. Potential applications within these include supporting better intervention design; developing toolkits of actions that can be used flexibly by practitioners with appropriate matching to young people’s needs and context; informing practitioner training; and refining existing practice (Ferber et al., 2019; McKaskill et al., 2021). An example of the latter has been developed in youth justice in the US (Lipsey et al., 2010) and found to be promising (Liberman & Hussemann, 2016).

### 2.3. Other Important Intervention Issues

#### 2.3.1. Engaging Youth and Families

Self-evidently, interventions to prevent and reduce youth crime and violence can only be effective if the people they target engage with them. Moreover, participation in psychosocial programmes is positively associated with positive effects. However, some groups of parents and CYP are less likely to attend interventions. Reasons for this intersect with social disadvantage: difficulty accessing interventions because of competing demands on time, work commitments or a lack of transport or digital means; issues with intervention acceptability, including social stigma, anxiety and a fear of being labelled a ‘bad person’; and a lack of awareness of interventions owing to, for instance, poor publicity or not appreciating the need for support (Finan et al., 2018; Pote et al., 2019; Clemmow et al., 2025a). Families already receiving services from multiple agencies may also struggle to engage with a new intervention.

There are many suggested strategies for better engaging parents in parenting and family interventions. These commonly address the aforementioned barriers, although evidence for their impact is limited (Finan et al., 2018; Pote et al., 2019). Additional effort is needed to engage socially disadvantaged parents: for example, through support with mental health (including strengthening sense of parenting self-efficacy); being encouraging, non-judgmental and patient; offering wraparound support; flexing to meet parents’ needs; and offering financial incentives (notwithstanding mixed evidence for their effectiveness) (Berry et al., 2023).

Efforts are also needed to support CYP access to and engagement with services to prevent youth violence. A recent review identified several barriers and associated strategies to address them (Clemmow et al., 2025a). For example, it helps to co-produce interesting and engaging activities around sports and arts, and to offer these in inviting environments. Ideally, these would be in local community settings, possibly co-located in universal services. Efforts to coordinate multi-agency support and offer a consistent single point of access for CYP are likely to help too. Practitioners need time and resources to develop relationships with CYP, and need to be caring, non-judgmental and attentive; this requires relevant experience and training. It may be helpful to have trained volunteers to support delivery, especially if there is distrust of statutory services. Additionally, engaging whole families can be helpful because parents can support CYP attendance and reinforce messages at home. It is essential to avoid language that is stigmatising or jargon-filled, and to take extra steps to include marginalised groups owing to, for instance, race and ethnicity or neurodevelopmental disorders. Of course, these strategies are likely to be connected, and more empirical research is needed to test their effectiveness.

#### 2.3.2. Multi-Agency Working

One significant barrier to providing effective services in this space is how poor multi-agency working is amongst agencies, certainly in the UK (Home Affairs Committee, 2019), which in turn can lead to services failing to identify CYP at risk (Maxwell et al., 2019). Perhaps most notable is the need for better multi-agency working between social care and the police, with different teams often taking a siloed approach, leading to CYP being handed over from, say, the police to social care and vice versa, with little joint working involved (Morgan, 2022). One of the most significant barriers is limited information sharing. For example, if a CYP known to social care is picked up by the police, that social care team may receive notification of the arrest, but this does not necessarily constitute a referral (Her Majesty’s Inspectorate of Constabulary and Fire Rescue Services, 2020) and, in any case, is not consistent practice. This, in turn, makes it very difficult to refer that CYP for appropriate CCE interventions that may already be available locally.

However, levers do exist to improve multi-agency working (Seekamp et al., 2022), which might provide the basis for pilot studies. For example, a pilot study evaluating new information sharing and joint working approaches^3^ across schools, the police and CYP social care is warranted. This would: (i) allow for better identification of at-risk CYP at an earlier stage; (ii) facilitate the delivery of prevention services; (iii) allow for agency involvement with CYP at points of crisis and reachable moments; (iv) facilitate referral for specialist services when CYP are found to be involved in violent gangs or organised criminal groups; (v) mediate police responses and allow for more trauma-informed and Child First^4^ approaches and less criminalisation; and (vi) allow for better risk assessment by social care teams and the provision of more appropriate and effective services.

#### 2.3.3. Adapting Programmes from Overseas

There is often a voltage drop in effectiveness when EBPs are replicated in new settings. Several US-origin youth violence and offending prevention interventions, such as FFT and MST, were not effective when tested in the UK (Humayun et al., 2017; Fonagy et al., 2018), and nor was the upstream social-emotional learning programme PATHS (Berry et al., 2016). Plausible theories for this phenomenon include faulty adaptation, poor fidelity, superior business as usual (the default control) in the new setting, and limited or no programme developer involvement (Green et al., 2023).

One course of action is therefore to seek to adapt programmes well. Adapting tested and effective programmes for new contexts is potentially more cost-effective than starting afresh. It involves the thoughtful and deliberate alteration of intervention design or delivery to improve its fit or effectiveness in each context (e.g., Evans et al., 2020; Movsisyan et al., 2021). Various frameworks and guidance exist to support programme adaptation (Stirman et al., 2019; Moore et al., 2021). Although this is potentially confusing, there is much consensus about principles (e.g., involving diverse stakeholders, agreeing a way of working, protecting the theory of change [ToC]) and steps (e.g., understanding the intervention and new context, consulting stakeholders, collectively agreeing changes). Most approaches advocate distinguishing between deep and surface adaptations, attending to intervention and context, and making changes before and during implementation.

There is a need for more evidence on the acceptability, implementation and outcomes of adaptation efforts, and to encourage better practice based on such evaluation. A recent narrative account of pre-implementation adaptation in the UK of a positive youth development programme from the US suggested that it helps if: (i) the adaptation team blends expertise in the intervention and local context; (ii) attention is paid to how implementation strategies in the original setting contributed to outcomes; and (iii) the intervention ToC is kept central and refined iteratively (Green et al., 2023). Some adaptation frameworks are overly academic and take too long to apply (Copeland et al., 2022), so they might be used more if simplified.

An alternative, but not mutually exclusive, explanation for why EBPs in the UK have not replicated US findings as often as expected is that the trial research methodology, and not just the intervention, needs adaptation, because the effectiveness of the methodology may be influenced by contextual factors. There are a number of factors to consider here, but the key ones are probably: (i) the counterfactual; (ii) factors affecting recruitment, which is related to (iii) differences in sample characteristics; and (iv) measuring outcomes (see Section 3.2 below). For example, RCTs of interventions will only answer the question ‘Is the intervention being trialled more effective than what we compared it to?’ As a result, it is important to consider whether failures to replicate the original effects of imported EBPs simply reflect superior business as usual (BAU) in the new host country.

However, it is likely that some of the factors that adversely affect trials attempting to replicate overseas EBPs are not fully reported due to challenges in publishing what may be quite descriptive data. As a result, it is likely that a great deal of learning in attempting to trial EBPs has not been shared. For future studies, evaluators might be encouraged to produce a secondary paper (e.g., Dixon et al., 2014), or a section in the project report, where there is more scope to discuss these issues. Naturally, these narratives would need to be interpreted cautiously but might provide useful information nonetheless.

## 3. Evaluation Issues

Here, we start by discussing common challenges in moving through the pipeline of feasibility, pilot and definitive (often trial-based) evaluations. We then explore issues concerning the actual design and conduct of such studies, before closing with thoughts on the potential value of underused (non-trial) methods of impact evaluation. The context for these reflections is twofold: first, the large number of trials yielding null, inconclusive or uninformative results, and the need to pre-empt this; and second, the unsuitability or even impossibility of using trial methodology for some forms of intervention, yet the need still to understand their impact.

### 3.1. Moving Through the Pipeline

#### 3.1.1. Preparation

Prior to commissioning or conducting any intervention evaluation, it is important to have a conceptual model outlining what is known about the subject area, covering outcomes, mediators, moderators and implementation factors (Axford et al., 2022b). This helps ensure that any proposed evaluation addresses known uncertainty. It should also inform measures and analysis and make it easier to consider how results contribute to the knowledge base. Additionally, evaluators need to work closely with providers and the funder initially to agree on numbers and a timeline and align recruitment efforts (especially given the challenges of contacting parents and vulnerable CYP). There should be upfront agreement with the funder and delivery team on measures to avoid difficulties later, such as providers objecting post hoc to the use of certain measures.

Care is also needed to ensure that the intervention is worthy of evaluation. Too often, post-mortems following null or negative trial results conclude that there were flaws in the initial programme design, or that adverse effects could have been foreseen (Axford et al., 2022a). One reason for this is that interventions often lack an established ToC. While developers may have a tacit ToC, this can often be at a very early stage and quite opaque. An understanding of the mechanisms involved might be based primarily on the experience of practitioners who have developed the intervention approach and less on well-established clinical theory.

One response to this challenge is to only consider funding feasibility studies when there is a fully articulated ToC. However, this is likely to result in a large number of promising interventions with a good track record of delivery being disregarded, as well as system level or practice models. An alternative is to build co-production of a ToC into the design of feasibility studies and for this to go hand in hand with the identification, or development, of primary outcome measures (see Section 3.2.1 below). In other words, funding for early feasibility work might incorporate funding for the evaluator to provide a scaffold for the development of a ToC and associated outcome measure(s). This could then act as a first, and quite early, stop–go criterion for the project, such that all funding ceases if a ToC and primary outcome cannot be characterised and identified. As a result, funding will not be wasted on conducting early-stage, but quite expensive, qualitative work assessing the acceptability of the intervention or proposed evaluation design or on a process evaluation study. This is an approach that the YEF has adopted.

The process of developing the ToC should be collaborative, involving practitioners, researchers, service users and the public. It should also be informed by theory and evidence, and iterative—a progressive ‘thickening’ of the theory. Basic standards to ensure that the ToC is tight include having a clear structure, carefully defined items and an explanation of connections. Potential adverse effects and means of averting them can be considered. A simple taxonomy of different types of possible harm may help promote reflection (e.g., Lorenc & Oliver, 2013).

#### 3.1.2. Capturing the Process of the Feasibility Study

Key information about intervention and trial feasibility can be captured by using formal process evaluation methodology frameworks (see Humphrey et al., 2016 for an overview), which are not used enough by evaluators. However, what tends to be missed, even in these frameworks, is some of the information captured during discussions and negotiations with key stakeholders. These usually occur outside of formal data capture processes but can constitute a large part of the feasibility study (for example, in a pilot evaluation of FFT (Humayun et al., 2023), there were 82 meetings) and are where many of the significant barriers to a trial emerge. Traditional qualitative methods are not necessarily best suited to such data (although some other qualitative methods are appropriate), so it may be worth encouraging Principal Investigators to provide a narrative as part of the write-up to better capture this information. This is challenging, because it involves a lot of data capture and there are research ethics issues to consider. However, this mechanism might allow for the capture of quite rich data that is typically lost.

#### 3.1.3. Moving from Feasibility/Pilot to Full Trial

Progressing from a small-scale feasibility study or pilot to a definitive (often large) trial can present significant challenges for intervention infrastructure and cost. For example, a youth mentoring programme operating in three schools in a pilot study might need to be delivered in 50 schools in a 100-school cluster trial. This would require the rapid and costly expansion of training and technical assistance. Delivery organisations do not necessarily aspire to this or appreciate it when embarking on feasibility studies. The possibility therefore needs to be factored in early on. This includes securing buy-in to that vision, exploring scalability potential within the feasibility study and considering trial designs that permit more gradual expansion. Stepped-wedge trials, for instance, involve staggering the start of intervention delivery (Hemming et al., 2015), although they extend trial length and still require simultaneous delivery in multiple sites, which may be prohibitive for delivery organisations.

#### 3.1.4. Towards ‘Thicker’ Definitive Trials

Trials of psychosocial interventions, including those concerned with preventing CYP crime and violence, often focus mainly on the difference in pre-specified outcomes between intervention and control arms. They might be characterised as ‘thin’. This is driven largely by funding imperatives, notably the desire to find out ‘what works’ on a limited budget. By contrast, ‘thicker’ trials are more substantial regarding the amount and nature of data collected, the analyses conducted and, sometimes, participant numbers. They are adequately powered, capture implementation fidelity, record regular services provided, and align follow-up data collection points with when outcomes are expected, even if these are long-term (Axford et al., 2022b). Mediator and moderator analyses help with understanding what works for whom and why (O’Rourke & MacKinnon, 2018), while Complier Average Causal Effect analysis unpacks the fidelity–outcomes relationship (Hewitt et al., 2006). Qualitative methods can help explain variations in outcomes, the mechanisms through which interventions generate impact, and why results might be disappointing or confusing (O’Cathain et al., 2014; Richards et al., 2019). Together, these approaches make trial results more informative. They also make trials more expensive, arguably necessitating fewer but better trials.

### 3.2. Evaluation Design and Conduct

#### 3.2.1. Outcome Measurement

We have addressed challenges in measuring outcomes for youth crime and violence prevention interventions elsewhere (Humayun et al., 2025) and highlight the key issues here only. Studies will only demonstrate the effectiveness of interventions if they demonstrate positive change in the targeted outcomes. However, intervention approaches at an early stage can often struggle to clearly identify their primary outcome because they may have been using minimal evaluation approaches and no well-established measures, or measures might be quite broad in terms of the outcomes they measure and fail to measure the specific outcome being targeted. Sometimes, a clear ToC has not been properly identified (see Section 3.1.1 above), making identifying appropriate outcomes challenging.

A common route to measure youth violence and offending involves using CYP self-completion measures before and after the intervention. However, this can result in very firm resistance from practitioners and CYP, and the quantity and quality of the data collected can be poor. Some alternative self-completion options seek to prevent the measures undermining the practitioner–CYP relationship. For instance, with the retrospective pretest, respondents are asked to report after an intervention on knowledge, attitudes or behaviours *before* the intervention (Little et al., 2020). An alternative to self-completion involves using data on administrative systems or collected by practitioners. These all pose significant challenges, however, such as the introduction of other biases in the case of retrospective data capture and practitioner-collected data, and incomplete recording or access difficulties in the case of administrative data.

Identifying appropriate outcome measures is typically less challenging when attempting to adapt EBPs from overseas. Quite often, outcome measures that are identical, or similar, to those used in previous trials can be adopted. However, there are three areas where outcome measurement may be problematic. First, when previous studies have used agency data as a primary outcome, the relevant outcome data may not exist, or be collected reliably, in the new setting. Second, the problem that the intervention is attempting to address in the new site might not be common in the country where it was developed, and so no established outcome measure exists. This has been the case in recent studies of FFT (Humayun et al., 2023) and MST (Langdon et al., 2023) for CCE and CLDN involvement. Third, attitudes to measuring the construct may differ significantly between locales.

#### 3.2.2. Recruitment

One potential explanation for failure to replicate EBPs is significant differences from the sample recruited in original effectiveness studies. While great care should be taken to ensure eligibility criteria match previous studies and are aligned closely with the intervention ToC, there are alternative explanations for why this might happen.

First, it may not be possible to screen effectively due to limitations in accessing administrative data, including barriers to signing an Information/Data Sharing Agreement with the site. In the YEF-funded FFT study, this required a huge investment in time from multiple stakeholders, and the process took two years (Humayun et al., 2023). Therefore, drawing up such an agreement should be near the top of the project priority list.

Second, referrals in the original RCTs may have been via a quite different route to what is possible in the new site. For example, a number of RCTs of EBPs in the US involve mandated engagement in services through family courts. This is hardly ever likely to be the case in the UK, which affects engagement by CYP and families, thereby impacting recruitment, and potentially resulting in a different sample.

Third, recruitment challenges can lead to eligibility criteria being relaxed and CYP who are not likely to benefit from the intervention being recruited. This problem is exacerbated in feasibility trials where it may not be entirely clear who will benefit the most, so some care needs to be taken in finding a balance between the competing needs of recruiting the right sample and adapting trial design as more is determined about the intervention and the population it is targeting. This is very difficult to achieve in a study that combines feasibility questions with a pilot RCT. The most viable route to achieving it is by clearly separating out the stages of research such that as much as possible is known about the ToC and the likely population it would benefit before assessing the viability of recruitment.

#### 3.2.3. Attrition

Attrition is as much a risk to the viability of an evaluation as recruitment problems. Factors affecting attrition are discussed extensively in the trial literature (e.g., Ramakrishnan et al., 2022) so will not be discussed in great detail here. However, it is worth noting two risks beyond the impact on overall power. First, missing data are not likely to be missing at random, because CYP with more severe problems are more likely to drop out, thereby impacting the generalisability of findings. Second, it is worth paying particular attention to factors driving attrition in control groups, where it tends to be higher and will therefore result in imbalance between groups post-treatment.

As a result, many clearinghouses assess whether trials have reported tests for equivalence between groups after attrition when rating the quality of evidence for interventions. While this addresses an important limitation, it does nothing to resolve it. What is more effective is capturing predictors of attrition at baseline and covarying for these in analyses. This requires a larger assessment battery (see above) but effectively resolves this problem unless very high rates of attrition have occurred.

#### 3.2.4. Business as Usual

RCTs of interventions answer the question ‘Is the intervention being trialled more effective than what we compared it to?’ However, we often know little about what business as usual (BAU, the default control) looks like. For example, in the first UK RCT of FFT (Humayun et al., 2017), the study team aimed to carefully record the quantity and nature of the counterfactual: the usual services provided by youth offending services. While some data on quantity was captured, it was not recorded reliably. The description of the services was via a choice from a drop-down menu, but 80% of cases were recorded as having received ‘miscellaneous’ intervention.

Understanding the nature of BAU is important for two reasons. First, it affects the size and interpretation of any effects observed. For instance, little or no effect (or even a negative effect) might be attributable to strong BAU. Second, there may be a great deal of high-quality service provision that is not manualised and branded and is therefore not considered worthy of trialling. It is worth considering conducting some scoping work to better understand what BAU looks like and explore whether there are approaches that would benefit from formal evaluation. It would also be good to have some sense of the effectiveness of BAU. One approach might be leveraging Network Meta-Analysis methodology to calculate effect sizes (Seitidis et al., 2022). This approach allows for direct comparisons between different intervention approaches that were tested in separate studies and is most often used to compare different pharmaceutical interventions. It might, instead, be used to compare control group interventions from separate trials.

#### 3.2.5. The Effect of Trial Methodology on Engagement

The idea that evaluation simply wraps around an intervention with no effect on user engagement in the intervention or on how the intervention is delivered is naïve. This is because recruitment to a trial might require multiple assessment meetings to be set up. Most importantly, it will require participants to complete an assessment battery when these families are likely to have already been heavily assessed by services.

Therefore, the experiences of CYP and families involved in an evaluation are often quite different to those just receiving the same evaluation outside of a trial. For example, in a recent evaluation of FFT (Humayun et al., 2023), the time from eligibility being determined to a family’s first meeting with a therapist was two days in the first (feasibility) stage of the study when there was no direct assessment of the families or process of recruitment to the study. In the subsequent pilot RCT, the time from determining eligibility to the first meeting with a therapist was 28 days.

There is not necessarily a solution to this problem, but it should be taken into account when interpreting the results of trials. This is particularly important when results are different in an early feasibility phase compared with a RCT phase. Under these circumstances, the assumption is that randomisation has impacted engagement. While that is often the case, it might just be that the sheer burden of trial involvement has impacted engagement.

### 3.3. Beyond the Pipeline Paradigm

The linear pipeline paradigm involves developing programmes and evaluating them through RCTs before scaling those found to be effective in improving CYP outcomes (Knox et al., 2018; Asmussen et al., 2019). It underpins the work of online clearinghouses and UK What Works Centres like Foundations and the YEF, and there is much to be said for the approach. It has contributed to the development of an extensive evidence base on what works to prevent youth crime and violence, manifested in multiple systematic reviews and meta-analyses (e.g., Farrington et al., 2017; Kovalenko et al., 2022). It has also produced a significant number of dissemination-ready EBPs to prevent or reduce crime and violence.^5^

However, not all interventions need to or can be evaluated experimentally. Reasons include a lack of resources, interventions not being ready for such scrutiny, and the absence of clinical equipoise. This presents challenges for efforts to prevent youth crime and violence. Specifically, the few interventions that reach the highest tier in online EBP clearinghouses are outliers; most interventions have no evidence of effectiveness or are left stranded in a no-man’s land between initial evidence of promise and full endorsement by a clearinghouse. Alongside identifying selected interventions that could be scaled or progressed along the pipeline (and helping them to do so), it is therefore imperative to improve the effectiveness of the many interventions that already exist. Part of this involves using alternative evaluation approaches to understand impact.

One set of approaches uses quantitative techniques to mimic control groups. These include the use of algorithms based on epidemiological data to estimate the added value of an intervention. For example, the Strengths and Difficulties Questionnaire Value-Added Score (Ford et al., 2009) has been used to evaluate FFT for high-risk CYP and their families (Marshall et al., 2018). Another method permits comparisons between pre- and post-intervention data on actual intervention participants on the one hand, and statistically derived controls based on government administrative data on the other (Adler & Coulson, 2016; Piazza et al., 2019). For instance, the Justice Data Lab assesses whether Ministry of Justice data suggest that changes in re-offending occurred due to a participating organisation’s offender rehabilitation work. Projects analysed this way include employment training and support and parenting support for fathers in prison.

There are also impact evaluation methods that rely more on qualitative data. These work by seeking to validate the intervention ToC or ruling out competing hypothetical causal mechanisms. For example, contribution analysis aims to compare an intervention’s hypothesised ToC against the evidence on what happened during and following intervention delivery to draw conclusions about its contribution to observed outcomes (Mayne, 2012). It focuses on the relative contribution of the intervention and its constituent components alongside other influences on participants’ lives, assessing convergence between different data sources to determine the strength of evidence underpinning different elements of the causal pathway. This approach was used in a pilot evaluation of the school-based youth mentoring programme Becoming a Man (BAM) to analyse 11 case studies, each one representing a BAM participant (Green et al., 2025). These were constructed by triangulating qualitative data from interviews with the BAM practitioner, the young person, their parent/carer and school staff regarding the young person’s journey through the programme, alongside quantitative data on programme implementation and participants’ social–emotional well-being and education. While BAM was generally ranked second or third behind other influences (e.g., family, friends, football), in line with the programme ToC, it supported CYP engagement in action and reflection, which in turn helped CYP to manage their emotions, bolstered their self-esteem and contributed to responsible decision-making (Green et al., 2025).

Place-based interventions can be challenging to evaluate owing to their size and complexity. Their evolving nature means that developmental and learning evaluation approaches may be helpful, as these involve feeding back and acting on early learning (Smith et al., 2023). In terms of measuring impact, certain types of experimental and quasi-experimental design may be suitable, including cluster trials, stepped-wedge designs, interrupted time series and propensity score matching, but there is also space for theory-based approaches that explore whether the intervention has plausibly contributed to observed changes (e.g., contribution analysis, outcome harvesting, Most Significant Change) (Smith et al., 2023).

Finally, there is a need for more evidence-informed improvement of existing provision (cf. Section 2.2.3 above). For example, the Standardized Program Evaluation Protocol for Assessing Juvenile Justice Programs approach in juvenile delinquency in the US involves: conducting a meta-analysis of relevant studies to identify elements most strongly associated with positive outcomes; systematically assessing regular practice against those elements to determine how far they are already implemented and identify areas for improvement; and supporting practitioners and managers to make changes to practice (Lipsey et al., 2010).

## 4. Conclusions

In this article, we have discussed key issues that need addressing to further the use of evidence-based approaches to preventing youth crime and violence. We have identified the need to pay more attention to hitherto neglected issues affecting CYP, such as ACEs, financial and material disadvantage, criminal exploitation and county lines, and argued for identifying interventions for CYP with CU traits. We have also advocated greater investment in different approaches to intervention design and delivery, such as teletherapy, place-based or collective impact initiatives and common elements. Other important intervention-related issues include the need for better engagement of CYP and families in services, improved multi-agency working and more careful adaptation of EBPs imported from overseas. The article additionally makes a series of recommendations for improving the evaluation of interventions to prevent youth crime and violence. Many of these are concerned with supporting the passage of interventions through the traditional pipeline model, from better preparing for evaluation and strengthening learning from feasibility testing, through smoothing the progression from feasibility and pilot studies to definitive trials, to designing full trials to be maximally informative. Several suggestions focus on strengthening standard evaluations (typically trials): for instance, by measuring outcomes more appropriately, improving recruitment, minimising attrition and better capturing business as usual (the default control). Finally, we argue that greater use needs to be made of alternative (non-trial) approaches to impact evaluation, particularly for interventions that are not programmatic in format. The issues discussed here are by no means exhaustive, and the recommendations no panacea; indeed, they necessarily reflect our respective experiences and judgement regarding what is important given the original brief. Nevertheless, we believe that attention to the issues raised and their potential solutions will improve efforts to prevent youth crime and violence through evidence-based practice.

## Data Availability

No new data were created or analysed in this study. Data sharing is not applicable to this article.

## Notes

1 https://www.gov.uk/government/publications/trauma-informed-practice-learning-from-experience/trauma-informed-practice-learning-from-experience-of-violence-reduction-unit-delivery-2021-to-2023-accessible (accessed on 22 January 2026).

2 For example, via CCE rescue teams: https://www.catch-22.org.uk/find-services/county-lines-support-and-rescue/ (accessed on 22 January 2026).

3 For example, the Operation Harbinger pilot of child in custody information sharing in several London boroughs: https://yjresourcehub.uk/child-in-custody-information-sharing-with-metropolitan-police-operation-harbinger-metropolitan-police-march-2021/ (accessed on 22 January 2026).

4 For an overview of the Child First approach see https://hmiprobation.justiceinspectorates.gov.uk/our-research/evidence-base-youth-justice/general-models-and-principles/child-first/ (accessed on 22 January 2026).

5 For example, the UK Foundations Guidebook lists 48 EBPs [searched 3 February 2026] targeting CYP aged 4 to 18 years for ‘preventing crime, violence and antisocial behaviour’. This counts programmes assessed as Level 3 and above, meaning that there is at least one rigorous evaluation showing a positive impact: https://foundations.org.uk/toolkit/guidebook/ (accessed on 22 January 2026).

## Abbreviations

The following abbreviations are used in this manuscript:

ACE	Adverse childhood experience
BAM	Becoming a Man
BAU	Business as usual
CCE	Child criminal exploitation
CLDN	County lines drug network
CU	Callous–unemotional
CYP	Children and young people/Child or young person
EBP	Evidence-based programme
FFT	Functional Family Therapy
MST	Multisystemic Therapy
RCT	Randomised controlled trial
ToC	Theory of change
YEF	Youth Endowment Fund
